# Consumer Perspectives on Processing Technologies for Organic Food

**DOI:** 10.3390/foods10061212

**Published:** 2021-05-27

**Authors:** Ronja Hüppe, Katrin Zander

**Affiliations:** Section of Agricultural and Food Marketing, University of Kassel, 37213 Witzenhausen, Germany; k.zander@uni-kassel.de

**Keywords:** processing technologies, organic processing, consumer preference, focus groups, food preservation, shelf life, consumer behaviour, organic food

## Abstract

Over the last years, consumer demand for natural and healthy convenient food has increased, and with it the demand for organic convenience food. With convenience food, the processing level increases, which consumers are sceptical of. This holds especially for organic consumers who prefer natural, healthy, and sustainable food products. In the literature, consumer preferences are investigated for processed conventional food, but rarely for organic products. Therefore, this study investigates consumers’ knowledge, expectations, and attitudes towards selected processing technologies for organic food. Nine focus groups with 84 organic consumers were conducted, discussing preservation technologies of organic milk and orange juice. Results showed that participants had little knowledge about processing technologies but were interested in their benefits. Organic processing technologies should include fewer processing steps, low environmental impact, while keeping the product as natural as possible. Since consumers want to know benefits but not details of processing, asking consumers for their specific preferences when developing new processing technologies remains challenging. This paper shows how consumers’ benefit and risk perception including their want for naturalness, and scepticism for new technologies shape their evaluation of (organic) food processing technologies. Two consumer groups with different attitudes towards processing could be identified: ’organic traditionalists’ and ‘organic pragmatics’.

## 1. Introduction

With a modern and fast changing lifestyle, consumers take increasingly less time for food shopping and preparation, especially in industrialised countries [1]. Therefore, convenience food is becoming ever more important [2]. In Germany, sales turnover of ready meals and soups increased constantly throughout the last years from 6.3 million euros in 2012 to 7.3 million euros in 2019, and peaked with 8.6 million euros in 2020, due to the Covid-19 pandemic [3]. With this increasing demand for convenience food, food processors have developed new food processing technologies to improve microbiological safety, taste, and shelf life [4,5,6,7] However, consumers are sceptical towards processed and convenience food and supposedly new technologies as they are often perceived as unhealthy, unsustainable, and unnatural [2,7,8,9,10]. 

Societal debates of health risks in foods caused by food scares, additives, and pesticide residues increase the interest in natural and organic food [11]. According to BÖLN [12], 78% of German consumers buy organic products at least occasionally whereas only 22% never consume organic products. Organic foods are often perceived as more natural than conventional products, and for many consumers, the naturalness of a food product is a key quality attribute, both for primary production and processing [10]. There is the desire for convenience on the one hand and for pure and natural foods on the other hand, challenging food processors to develop adequate food processing technologies [13,14]. This holds true especially for organic consumers, since they tend to prefer traditional and natural processing technologies [15], but they also increasingly demand organic convenience food [16].

Looking at the literature, there is a body of research which deals with consumers’ risk and benefit perceptions (see e.g., [17,18]) as well as with food technology neophobia (see e.g., [7]) and naturalness perception of processed conventional food (see e.g., [19,20]). Yet, to our knowledge, only Asioli et al. [21] and Popa et al. [22] studied consumer preferences regarding organic products. 

Consumers perceive organic products often as natural and minimally processed [23,24] and natural products are often characterised as minimally processed and organic [25]. They associate similar benefits with natural and organic food [26], and used the terms ‘natural’ and ‘organic’ interchangeably when web-searching to purchase organic food [27]. Moreover, consumers were found to have no significantly different willingness to pay for either organic or all-natural products indicating a similar understanding of the terms [28]. 

It is known that consumers of organic food have strong environmental and ecological values [29], which leads to the assumption that they tend to have different expectations of food processing than consumers of conventional food do have. Therefore, understanding consumer preferences for processed organic food products will reveal new insights into the preferences of organic consumers and the acceptance of technologies used for processing organic foods. It can help food processors to develop adequate technologies and reduce the risk of losing market shares if consumers do not accept their technologies. In particular, this study investigates consumers’ knowledge, expectations, and attitudes towards selected processing technologies for organic food based on the following research questions: ’What do consumers know about (organic) food processing?’, ’What do consumers expect from organic processing?’, and ’Which of the presented processing technologies do consumers prefer for organic food?’. For this purpose, an exploratory qualitative research was conducted with buyers of organic food.

This article is divided into six sections. After an introduction, the second section gives an overview over the relevant literature regarding consumers’ attitude formation and concepts that influence food technology acceptance: food technology neophobia, risks and benefits of food processing, and the perception of food naturalness. A special focus is laid upon non-thermal technologies regarding shelf life. The third section describes the qualitative study design. The results are presented in the fourth section which is followed by the discussion in the fifth section. This article closes with some conclusions in the sixth section. 

## 2. State of the Art

For consumers, the main reasons to buy organic food are health concerns, product quality, and concerns regarding nature and environmental degradation [29]. Besides, reasons such as better taste and animal welfare are also known to be important [30]. 

Also in regard to processing, organic food consumers are more sensitive to and prefer natural, healthy, and environmentally sustainable food products [21,22]. For example, Popa et al. [22] looked at preferred attributes for organic berries on packaging in Romania They found that consumers preferred air-dried Romanian berries with higher nutritional content, instead of microwave-dried berries from Europe. Asioli et al. [21] compared air drying (thermal) with microwave drying (non-thermal) for organic strawberries. Consumers who cared about organic, natural, and environmentally friendly products were particularly sceptical of new technologies and therefore preferred traditional air drying. It was perceived as more natural, although microwave drying consumes less energy and preserves nutrients and flavour better [21]. Thus, organic consumers seem to perceive risks and benefits as well as naturalness more sensitively while preferring traditional processing technologies [15].

One of the main requirements for organic processed food is the use of organic ingredients. In addition, the Codex Alimentarius requires to process organic foods with careful processing methods, reducing the use of additives and processing aids as much as possible. The use of additives is regulated by ’positive lists’ [31]. The standards of the International Federation of Organic Agriculture Movements (IFOAM), define organic processing as making use of biological, mechanical, and physical processes, keeping the ’true nature’ of the product and ensuring sustainability for the environment [32]. That means there are some clear guidelines that must be followed and there are concepts, such as ‘careful processing’, that should be considered when processing organic food. However, there is neither a clear definition of what ‘organic processing’ is, nor is there one for the terms ‘true nature’ or ‘careful processing’ [24,33]. 

Consumers vary widely in their perceptions and attitudes towards food processing technologies [34]. Attitudes are formed over time and can be explained by two complementary approaches: bottom-up and top-down, depending on the knowledge consumers have about a food processing technology [35,36]. In general, consumer awareness and knowledge of food technologies is low [17,18,36,37]. When costs and benefits are not known, consumers follow higher-order values and fundamental beliefs and hence, a top-down approach. If consumers know benefits and costs of a technology, they decide based on a functional rationality that is bottom-up [36]. Troy et al. [37] found that consumers are often not interested in developing an information-based bottom-up approach for building their attitude towards a new technology. They rather rely on their own perception and hence, on a top-down approach. Similarly, Barcellos et al. [38] found that consumers want information about the benefits of a new technology but no details on what happens inside the processing plant. Siegrist and Hartmann [20] described decision making in the absence of knowledge as the affect heuristic, which is the affective meaning, an association, or narrative people rely on when evaluating a technology. The affect heuristic might also be an important reason why people judge the same technologies differently. 

One important concept is “food technology neophobia” (FTN) which is used to explain consumers’ attitudes towards new food technologies and their willingness to try products which are processed with pre-existing or new technologies [7,39,40]. The FTN scale helped to improve the predictability of consumer behaviour regarding food choices of different highly processed familiar foods [40]. Based on the level of affinity towards new technologies, consumer segments were built. Consumers who were rather sceptical and traditional had concerns that new food processing technologies would negatively impact food naturalness and the environment as well as health and quality of life [41]. Indeed, consumers who had high levels of FTN rated fruit juices more negatively than consumers with low or medium levels of FTN, regardless of whether juices were produced with conventional or new technologies [7]. Consumers who generally tend to have strong environmental and ecological values also tend to have higher levels of FTN. They are more reluctant to try new technologies, although the new technology might be more environmentally friendly [21]. 

Moving on to consumer acceptance of food processing technologies, perceived benefits and risks are two influential concepts. They mostly concern healthiness, nutritional quality, food safety, and the environmental impact of a food product and differ between people [34]. The acceptance of food processing then depends on how obvious benefits are to consumers [18], and if they can recognise personal benefits, such as more healthiness and eating quality [38]. Moreover, acceptance depends on whether consumers can control their exposure to a technology, and the potential ethical or environmental impacts of the latter [17].

Due to the increased interest in healthy and natural foods, ‘food naturalness’ also influences consumers’ perception and acceptance of processing technologies; during primary production, processing, and in the final product [7,10,19]. For the majority of consumers, naturalness has a positive reputation [20,25]. This has also been described as the ’natural-is-better’ heuristic [20]. Healthier, better tasting, and environmentally friendly were the main positive aspects of the natural-is-better heuristic. These aspects did not only influence consumers’ acceptance when evaluating technologies, but also had the potential to bias technology evaluation [18,20]. Hence, it is important to understand how interventions and technologies influence consumers’ perceptions of naturalness. Consumers associated with naturalness the absence of intervention or changes and hence, processing was generally perceived as decreasing naturalness [38,42]. Chemical changes were assumed to decrease naturalness of a product more than physical changes [42]. Dickson-Spillmann et al. [43] even talked about a ‘chemophobia’ where consumers deemed something natural as safe and associated something chemical or synthetic with toxic. 

Looking at specific technologies, such as high-pressure processing (HPP) and pulsed electric fields (PEF), benefits are improved nutrient conservation and taste, and also less energy consumption, compared to thermal technologies (e.g., pasteurisation) [44,45]. In general, consumers perceived risks of high-pressure processing (HPP) as rather low. Benefits such as taste, nutritional quality and environmental friendliness seemed to be clear, and exposure to and extent of the technology well manageable [17,46]. Moreover, Sonne et al. [45] found out that consumers preferred HPP technology due to its high product quality and environmental friendliness and associated it with two important values: health and ’being good to nature’. 

Compared to PEF, HPP was perceived more positively. PEF contains the term ’electric’ which consumers associated with ’electricity’ and hence, with something negative in relation to food [46]. When compared to cold-pressed or fresh juice, consumers had implicit fears regarding HPP-treated products and rather negative associations [7]. The main barrier to increase acceptance of HPP was lack of information and lack of trust in the food industry [18,46]. To overcome this, technology information should be communicated via a product label [45]. Here, the main perceived benefits of HPP should be communicated in a direct and easily understandable and traceable way [34]. Summing up, HPP has many benefits which are recognised by the consumer. However, the general knowledge on processing technologies is rather low. Hence, obtaining further insights into risk and benefit perception of HPP for organic products is necessary.

Concerning the perception of naturalness, HPP and PEF technologies were seen as more natural–compared to pasteurisation–since they preserved a fresh taste and nutrients [45,46]. When compared to fresh or cold pressed juices, HPP treated juices were seen as less natural and the food industry has even promoted products as ’HPP free’ [7] (p. 2). In summary, the naturalness perception of technologies differed depending on consumers’ heuristics about the technology, as well as the comparative products or technologies.

## 3. Materials and Methods

### 3.1. Focus Groups

Since little prior research exists regarding consumers and processing technologies of organic food, focus groups (FG) as an explorative method were chosen to collect primary data. FGs are carefully planned discussions with about six to ten participants [47]. The discussions follow a structured but variable set of guiding questions which allows adapting to the natural flow of the discussion [48]. This also means that questions aim to stimulate the discussion rather than asking for specific reasons. This motivates participants to mention the aspects they consider important for the topic at hand and enables the researcher to get the participants’ viewpoint [49]. Moreover, the participants can probe each other’s responses, which allows the researcher to get deeper insights about participants’ reasoning and social interactions [47,50]. In addition, so-called heterogeneous and ad-hoc groups in which participants meet different socio-economic criteria and do not know each other before the discussion, lead to more lively and controversial discussions [51]. This enables the researcher to get diverse insights into a topic, a wide array of opinions, and a better idea of participants’ understanding, also of complex topics [49,52]. Besides their explorative character, FGs usually create a natural and relaxed atmosphere, reminding participants of an everyday communication situation. This encourages participants to express their honest opinions, enhance mutual learning, and hence allows obtaining rich, content-related, and realistic results [50,51]. However, if some people are very talkative or have strong opinions, others may remain silent and their opinion is lost. This can push the discussion in a certain direction and can bias the results. Therefore, the moderator should aim to balance these dynamics and ensure that every participant contributes her or his opinion [47]. Usually more than one focus group for each topic under investigation is conducted, ideally until the point of theoretical saturation is reached. This ensures consideration of the entire spectrum of opinions [47].

### 3.2. Study Design

In this study, nine focus group discussions were conducted with a total of 84 participants: five groups in Germany and four groups in Switzerland with 8–11 participants for each group. The sample consisted of consumers who bought organic products at least once in two weeks, in order to avoid participation of consumers who are reluctant to buy organic food. Participants were recruited via a market research agency with the following socio-economic criteria for each focus group:

- 33–66% female;
- 50% between 18 and 45 years and 50% between 46 and 75 years of age;
- a minimum of 33% and a maximum of 66% employed full or part-time.

In order not to overstrain the participants, well-known test products with a low processing level were chosen: milk and orange juice with a focus on processing technologies concerning shelf life.

The FGs were conducted in March 2019 by the first author and lasted 90 min. 

The focus groups followed a semi-structured discussion guide with main and subthemes, and corresponding questions. The discussion started with a short introduction round. As an introduction to the actual topic, participants were asked for their associations with processed foods, and for their ideas of advantages and disadvantages of processed food and food processing. Participants were then asked to discuss their expectations of processed organic food and how organic processed products differed from conventional processed products. 

In the main part of the discussion, specific processing technologies were discussed, starting with the example of milk. Participants where first asked what kind of milk they bought and why. Then different milk packages were presented. Afterwards, some information was provided about homogenisation and participants’ associations were discussed. The next topic concerned preservation technologies. After asking participants how long milk should last, some information on specific preservation technologies were presented. Participants discussed their associations, the compatibility of the technologies with their idea of organic as well as their technology preferences for organic milk.

Thereafter, orange juice was discussed, following a similar structure. First, a package of juice from concentrate was presented and the ingredients and claims were read to the participants. Then they were asked about their spontaneous thoughts concerning orange juice from concentrate. Following this, the processing steps for direct juice and juice from concentrate were presented and participants preferences for organic juice were discussed. An overview of different preservation technologies was presented to the participants: pasteurisation and high-pressure preservation as well as fresh juice as an example of minimal processing. Again, participants’ associations and the compatibility with their idea of organic processing was discussed and preferences for organic juice processing were identified.

### 3.3. Data Analysis

The audio recordings were fully transcribed by a professional transcription agency. The analysis followed a thematic qualitative text analysis [53]. First, the transcripts were read through to get an overall impression. Then, categories were built according to the main topics of the focus group structure–prior knowledge, expectations of processed organic food, milk processing technologies, orange juice processing technologies, and careful processing–and coded accordingly. Afterwards, subcategories were built, either as more detailed aspects of the main categories (deductive) or derived directly from the text (inductive). Subcategories included e.g., associations with a specific processing technology, preferences for processing technologies of organic milk or orange juice, and topics emerging from the discussion, such as trade-offs between convenience and quality. Since no major differences were found between the German and the Swiss FGs, both groups were analysed together. The software ‘MaxQDA Standard 2018’ [54] was used for building a category system and coding.

## 4. Results

From the introduction round of the discussion, it became clear that participants varied widely regarding their purchase behaviour of organic food and the range of products they bought.

### 4.1. Associations of Processed Food and Expectations of Processed Organic Food

Processed foods were primarily associated with concepts such as additives, artificial flavours and preservatives, E-numbers, chemicals, and packaging waste. These concepts often had negative connotations and stood in contrast to ’natural’.

“[With processed foods] I also associate something unhealthy, too much sugar, too much salt, too much fat, and also all these E-numbers.”

(FG B_2, P3)

Perceived advantages of processed food were time savings, convenience, easy portioning, and the ability to consume a wide variety of non-seasonal products.

“The advantages of processed food are also when you are lazy. You buy it ready made and you simply have to heat it up, for example.”

(FG CH_2, P2)

Although participants listed many different aspects, special processing technologies, such as freezing and pasteurisation, were rarely mentioned. Moreover, participants mentioned a general insecurity concerning processed food and the multiple aspects related to it, e.g., ingredients, packaging, or CO_2_ footprint.

“Right now, I read a lot in the media. But then what I am reading, then I thought, oh, now I do it right and then, I read another article and then it is wrong again. So, sometimes I am so overwhelmed with the diet and with the purchase.”

(FG B_1, P9)

After having discussed processed food in general, the discussion was focused on organically processed food. The perceived positive aspects of processed food also held true for most participants for processed organic food: time-savers, convenient, and easy to portion. Participants expected processed organic food to have rather few and organically produced ingredients, and to be mostly healthy and locally produced. They expected no or fewer additives, artificial flavours or preservatives, and as few ingredients and processing steps as possible.

“[…] when I buy an organic product, I always think, it has as few ingredients as possible, it is as natural as possible.”

(FG B_1, P3)

“[…] for such an organic product I expect the list of ingredients to be shorter than for a conventional product […].”

(FG B_2, P8)

Processing technologies were mostly not part of participants’ concept of ‘organic’ and thus, rarely mentioned. For most participants, ‘organic’ meant raw products from rather small-scale agriculture with a focus on animal-welfare and plants grown without pesticides and fertilisers.

“Well, it matters little how [the milk] is heated afterwards, whether it is organic or not. It depends on the origin of the product; how the cows were fed, which kind of medicine they got, and not on the heating method.”

(FG CH_1, P11)

“I expect a mindfulness in the production of organic products and appreciation towards the animals and also the products […].”

(FG B_1, P1)

Thus, organic products were deemed to be more valuable and natural from the outset compared to conventional products and should be processed in a way that keeps this value. 

Participants perceived the topic as very complex and often digressed from discussing expectations of processed organic food to what ‘organic’ meant to them. They were aware that their ideal of ‘organic’ deviated from reality and thus, their purchase behaviour in daily life varied as well. Furthermore, transparent and sustainable value chains were associated with organic products. In some cases, plastic packaging, little information about sustainability and lack of transparency, e.g., in product declarations, unclear origin–‘you don’t know where it comes from’–and a high CO_2_ footprint due to long transport routes caused mistrust in the organic sector. Hence, participants clearly associated transparent and environmentally sustainable value chains with organic foods which, for some, were even more important than the processing technology itself. 

### 4.2. Preferences of Processing Technologies for Organic Foods

First, specific processing technologies for milk were discussed. Information about homogenisation (Box 1) was provided with the following wording:

Box 1 **Information given to participants about homogenisation of milk based on Strahm and Eberhard [55].**

During homogenisation, the milk is forced through a nozzle under high pressure, which results in an even distribution of fat particles in the milk, preventing creaming.

Whether milk should be homogenised or not seemed to be mainly a question of habits and age. Older participants preferred non-homogenised milk because they knew it from their childhood, or they preferred a more natural product. Homogenisation of organic milk, as a mere physical treatment, predominantly corresponded to most participants’ perception of ’organic’ because nothing was added.

“When pressed through a machine it is still organic milk.”

(FG CH_3, P6)

Concerning shelf life, pasteurisation, microfiltration, and ultra-high temperature treatment, the following information (Box 2) was given to the participants:

Box 2 **Information given to participants about preservation technologies for milk based on Boitz and Mayer [56] and Strahm and Eberhard [55].**

During pasteurisation, the milk is heated to 72 °C for 15 s and bottled. Cooled, it has a shelf life of seven to ten days and is referred to as traditionally produced fresh milk. During microfiltration, skim milk is microfiltrated and the cream pasteurised at 125 °C. The two components are then mixed together and pasteurised again at 72 °C for 20 s. The microfiltered milk is declared as “fresh milk-longer lasting”, has similar good nutritional values as pasteurised milk and keeps refrigerated up to 21 days. In the ultra-high temperature (UHT) treatment, the milk is heated to 135–150 °C for three seconds. So-called UHT-milk has a “cooked” taste and fewer valuable ingredients than pasteurised or microfiltered milk and lasts at room temperature for up to several months.

Attitudes toward preservation technologies differed among participants. For some, processing was a prerequisite for making milk tradeable, while others were more sceptical about processing or separating and recombining the milk components. Pasteurised organic milk, as a long-established product, was accepted by the participants. For some participants, it was the most natural and freshest product and thus, the only choice.

For other participants, extended shelf life (ESL) organic milk, i.e., microfiltered fresh milk labelled ’longer lasting’, was a good alternative to pasteurised milk. However, participants did not agree with the term ’fresh milk’ for ESL-milk, but still associated it with a fresher taste and preferred it to UHT milk. 

Others needed only small amounts of milk and preferred ESL milk to prevent spoilage and thus, food waste. For some critical regular organic consumers, ESL milk was too highly processed and therefore did not meet their understanding of organic food. UHT milk was discussed controversially. For most participants, neither nutritional benefits were clear, nor was the technology perceived as careful or natural.

“[...] I prefer this ESL milk, because I always associate organic milk with an aspect of freshness. And that’s exactly what UHT milk doesn’t have, this freshness, that’s what I miss about it.”

(FG HH_1, P7)

Others accepted UHT milk out of habit or convenience.

“I actually think it’s quite good [that there is organic UHT milk], because for me organic doesn’t necessarily have anything to do with the freshness, the quality, in case of animal products, but rather how the animal was kept, as a difference. And if there is UHT milk, then it should rather be in organic quality, if the consumer wants to buy it.”

(FG HH_1, P2)

With a fast-moving lifestyle and milk being a highly perishable food, participants faced a trade-off between convenience and quality. For most of the participants it seemed to be a minor conflict. Many participants stated to simply choose conventional products when the organic ones did not entirely fit their needs.

Following milk, juice from concentrate in comparison with direct juice as well as preservation technologies for orange juice were discussed. First, the processing steps of direct juice and juice from concentrate (Box 3) were explained:

Box 3 **Information given to participants about processing steps for direct juice and juice from concentrate based on Schobinger [57].**

Direct juice is pressed, deep-frozen, pasteurised and bottled. For juice from concentrate, flavours and water must first be removed from the juice and the concentrate must be transported frozen. For re-dilution, water and flavours must be added, and the mixture must then be pasteurised and bottled.

Juice from concentrate elicited a spontaneous negative reaction from some participants.

“Well, if it [juice] is concentrated, then I don’t need that at all […].”

(FG B_2, P3)

In addition to the high energy and water consumption, the many processing steps, loss of nutritional value, supposed addition of extra sugar and flavourings, origin of the fruit, production conditions, doubts about the addition of water when re-diluting the concentrate, or simply general distrust, were reasons for not buying. Many participants preferred direct organic juice. Some participants even preferred conventional direct juice to organic juice from concentrate. Others also commented favourably on juice from concentrate and highlighted the equally good nutritional values and environmental benefits of transporting concentrates versus juice or whole fruits.

“For transportation it’s pretty good. If you transport it [concentrate] without all the water, you can transport a lot more!”

(FG HH_2, P3)

For some, taste and fruit content or origin of the fruit from organic farming was more important than processing technology. Fewer processing steps were also associated with more careful processing and consequently direct juice corresponded to the organic idea of many participants.

Following this, the preservation processes for orange juice, i.e., fresh juice, pasteurisation, and high-pressure processing (HPP), were further explained (Box 4).

Box 4 **Information given to participants about preservation technologies for orange juice based on Timmermans et al. [58].**

Fresh juice is pressed, bottled and has a refrigerated shelf life of up to seven days. High-pressure processed (HPP) juice is bottled in PET bottles and subjected to 6000 bar pressure in a water bath to kill harmful microorganisms. Vitamins are preserved and shelf life is extended to 21 days. Thermally pasteurized juice is heated to 80 °C and can be stored at room temperature for several months. Heating decreases the vitamin content and the juice is less aromatic.

Participants preferred fresh organic orange juice, although the shelf life of only seven days was a challenge for some. Therefore, participants were generally very positive about 21-day shelf life HPP-juice, although the technology was new to most of the participants. They did not perceive the necessary high pressure as problematic as long as nutrients were preserved and shelf life increased.

“So, I’m excited [about HPP] because I only buy fresh [orange juice], I love it, it tastes great, but, if I can make it longer durable and not have any vitamin loss, perfect!”

(FG B_1, P10)

Some had concerns about potentially high energy consumption and the use of PET bottles.

“But a PET bottle for organic juice?”

(FG HH_2, P10)

Moreover, some participants associated longer shelf life with less food waste.

In summary, consumers preferred direct and fresh organic orange juices due to good quality and naturalness perceptions. High-pressure processing was predominantly accepted or even preferred over pasteurised juice when it is better for the environment and a longer shelf life and thus, convenience was preferred. Whether HPP or pasteurisation was the more careful technology was not clear.

## 5. Discussion

Consumers had little awareness of processing technologies and rather limited knowledge of the technologies discussed in this study which is corroborated by the literature [9,18,19,37]. Processing technologies were mainly not part of their ’organic idea’. Consumers perceived processed organic products rather negatively in terms of quality and as the opposite of ’natural’, but they saw also positive aspects of processed organic foods, especially regarding convenience. Consumer preferences for specific processing technologies differed, depending on lifestyle and habits. Where some consumers preferred the least processed and natural product possible, pasteurised and unhomogenised milk, others valued ESL milk and convenience, which fitted their lifestyle and still had some amount of freshness to it. Highly processed products, such as UHT milk, were often seen as not compatible with organic processing. 

### 5.1. Attitude Formation and Heuristics

Findings suggest that consumers form attitudes both bottom-up and top-down, depending on their perception, knowledge and interest in a specific processing technology. For complex issues and little prior knowledge, consumers decide mainly based on higher-order values (top-down) (see also Veflen Olsen et al. [36] In this study, general values and hence, a top-down approach rather than a knowledge-based rationale, appeared to drive perceptions of most of the processing technologies for organic foods presented here. Troy et al. [37] also found that consumers are not interested in gaining detailed knowledge on food processing technologies. They prefer to rely on their own perceptions and values. 

Looking at the formation of attitudes in more detail, it depended on how easy technologies were to understand and on the type of product. For example, as higher-order values of organic consumers also include ethical aspects, such as animal welfare, when evaluating organic products [59,60], a top-down approach may influence the evaluation of animal products more than the evaluation of plant products. Indeed, when evaluating preservation technologies for milk, the ethical raising of the cows was often more important than the processing of the milk. On the contrary, in the example ’direct juice or juice from concentrate’, consumers followed a rational bottom-up approach (see also Scholderer and Frewer [35]), evaluating the technologies based on functional characteristics, such as number of processing steps, and perception of nutritional quality. The primary production of oranges was less important. This was in line with Melovic et al. [60], where organic consumers evaluated organic products based on their functional characteristics. 

Regardless of whether consumers follow a bottom-up or top-down approach, when knowledge is low, simple heuristics can also guide the evaluation of a technology [20,61]. In this study, complexity of evaluating processing technologies for organic foods was often reduced to ’natural-is-better’, ’fewer ingredients’, and ’fewer processing steps’. This became clear when comparing the preferences for juice from concentrate and direct juice. Most participants preferred direct juice as a processing technology for organic food. The fewer processing steps, the taste, and the nutritional quality increased the perception of naturalness compared to juice from concentrate. For the latter, lower perceived nutritional quality and the many processing steps reduced participants’ naturalness perception. Here, the natural-is-better heuristic [20] may have led participants to the preference of direct organic orange juice. However, some saw the transportation of concentrate as a benefit for the environment and also preferred organic juice from concentrate. 

### 5.2. Food Technology Neophobia and Perception of Risks and Benefits

The acceptance of a food technology further depends on consumers’ general level of food technology neophobia (FTN), and on the perception of risks and benefits of a technology. If benefits and risks are obvious to consumers, technology acceptance increased [18]. Asioli et al. [21] found that consumers with strong environmental and ecological values also tend to have higher levels of food technology neophobia (FTN). Higher levels of FTN lead to non-acceptance of both traditional and new technologies, as was found by Martins et al. [7]. Indeed, in this study, some critical organic consumers were identified and seemed to have an aversion towards processed organic food in general and towards new processing technologies in particular. These consumers did not perceive any benefits of processing beyond the minimum requirements and strongly valued natural and unprocessed organic products and traditional and familiar processing technologies. For them, the processing of organic foods was mainly associated with risks, such as loss of naturalness, loss of nutritional value, and negative health and environmental impacts, which was also found by Sajdakowska et al. [41], yet studying conventional products. Accepted technologies for organic processing were homogenisation and pasteurisation, where risk perception was rather low, benefits were clear and the technologies traditional and familiar (see also Barcellos et al. [38]). 

Besides these critical organic consumers, more tolerant organic consumers were also identified. They may also have strong ecological and environmental values [62], but compared to the critical consumers, they valued some benefits of processed organic foods and new processing technologies. These were increased shelf life, nutritional quality and health, naturalness, and potentially low environmental impact. The perceived risks were negative environmental impacts or packaging of products. Rather than fearing risks, these consumers often faced a trade-off between convenience and quality. For example, as an alternative to UHT milk, microfiltrated ESL milk was perceived as more natural since nutrients and a fresh taste were retained. For these consumers, microfiltration qualifies as a careful or more natural processing technology compared to UHT and thus, as a quality indicator for organic processing. As an alternative to pasteurised milk, the benefits of a longer shelf life and hence, increased convenience and potentially less food waste, outweigh the ‘risks’ of decreased naturalness and nutritional quality. Similarly, comparing HPP to pasteurised juice, benefits were a fresher taste, and a higher level of nutrients, which led to a higher level of perceived naturalness [45,46]. Consumers who compared HPP to fresh juice, appreciated the longer shelf life and associated it with the reduction of food waste [34]. The necessary PET-bottles and the presumably high energy consumption, in contrast, were perceived risks [34], while the high pressure was of no concern. Participants who saw HPP juice as an alternative to pasteurised juice, clearly preferred it as a careful and organic processing technology. Generally, these participants seemed to be more open towards more highly processed organic products and possibly also towards new technologies.

The more tolerant organic consumers also mentioned that ‘longer lasting’ milk should not be called ‘fresh milk’, as they expected transparency, especially for organic products. Song et al. [34] also identified transparency as an important consumer desire when purchasing food, yet studying conventional products.

### 5.3. Perception of Naturalness

The acceptance of a technology also depends on the perception of naturalness [7,10,19]. Since naturalness is closely related to organic food [25,26], this aspect becomes even more relevant for the evaluation of processing technologies for organic foods. Organic food should be processed ‘without additives’ if possible or contain ‘as little of something as possible’. Similarly, Román et al. [10] found that consumers associated ‘natural food’ with ‘no additives’ or ‘unprocessed’. Concerning the risk of losing naturalness, homogenisation as a mere physical treatment was accepted for organic milk as it seemed to decrease naturalness less than chemical changes do; this was also found by Rozin et al. [42]. Microfiltration as a processing technology for organic ESL milk was perceived to be too processed and unnatural and hence, not acceptable for organic processing by critical organic consumers. The same holds true for organic HPP-treated orange juice. The participants compared HPP to fresh juice and perceived the latter as more natural and careful than HPP and saw the higher processing level as a risk to the nutritional value. Moreover, other authors found that the naturalness perception of HPP [7] or generally non-thermal technologies [34] decreases when compared to fresh or cold pressed juices. Another reason might be consumers’ increasing interest in healthy foods, and thus their general aversion towards processed juices [13]. 

The general preference for natural products may also increase due to the Covid-19 pandemic which changed consumers’ behaviour towards more healthy and balanced diets [63]. However, with an easing pandemic, consumers are also likely to go back to old patterns [64], which indicates that the present results give still valuable insights and may even gain importance against the background of an increasing consciousness for healthy diets. 

### 5.4. Limitations

The research presented here is the outcome of focus group discussions. It gives some initial insights into consumers’ knowledge, opinions, and expectations regarding processing technologies for organic food. Due to the inherent character of the focus groups, that is qualitative and explorative, the findings are not representative, however, they are supposed to give a good overview of consumers’ perceptions and attitudes. The specific challenge in this research was that participants had only limited knowledge of the research topic of food processing technologies. This was accounted for by providing information about the technologies within the discussion. The information aimed to be as objective and neutral as possible, but an influence of the discussion as opposed to no information provision is likely and desirable since it enables a common basis for discussion. 

## 6. Conclusions

This study gives new insights into consumers’ perception of processing technologies for organic food. Although consumers have little knowledge regarding processing technologies and do not want to become experts of processing, they are still interested in the benefits they get from (new) processing technologies. Based on the results of this study, (organic) consumers mainly apply more general values when evaluating processing technologies. 

Two groups of consumers seem to emerge from the discussion: the ‘organic traditionalists’ and the ‘organic pragmatics’. The ‘organic traditionalists’ follow a top-down approach, guided by strong ecological and environmental values and a general scepticism to higher processing levels and processing technologies. Hence, organic products should be carefully processed in the sense of keeping the natural value and changing the product as little as possible; e.g., pasteurised milk, or direct and fresh juice. They also perceived none of the discussed processing technologies as careful. For these consumers, the entire value chain is important, including the processing stage. Organic processing should actively communicate the benefits of new technologies, such as fewer processing steps, good nutritional values, and lower environmental impacts and energy use, and in doing that, increase transparency for consumers.

The ‘organic pragmatics’ follow a bottom-up approach, valuing the organic primary production and convenience more than careful organic processing. The latter is ‘nice-to-have’ but not a decision criterion of whether an organic product should or should not be bought. They also accept higher processing levels for organic products because they see benefits in e.g., a longer shelf life (ESL or UHT milk), environmentally friendly transportation (juice from concentrate), or better nutritional values with longer shelf life (HPP-treated orange juice). For these consumers, the organic primary production is an important decision criterion since the processing technology itself is not always relevant for their evaluation of an organic product. Nevertheless, health and environmental benefits of new technologies should clearly be communicated to outweigh potential risk perceptions.

Consumers have different preferences and understandings concerning processed organic food and organic food processing. They want to know the benefits of a (new) technology but not the details of processing, which makes it difficult to include consumers in developing new (organic) processing technologies. 

Organic food processors should follow a holistic approach to organic processing, taking consumers’ values into account in order to prevent disappointment of consumers’ expectations of organic processing. Sound, open and honest communication of processing technologies should be part of any sustainable, and transparent organic value chain. Not only food processors, but also the organic sector in general could benefit from taking a leading position in transparent consumer communication. 

This research has shown that the theoretical concepts of bottom-up and top-down attitude formation, food technology neophobia, benefit risk evaluation and naturalness are helpful to better understand and predict consumers’ acceptance of food processing technologies. Hence, characterising different organic consumer segments regarding the general acceptance of organic processed food could give further insights. A follow up quantitative study could reveal the relevance of the different theoretical concepts for the acceptance of processing technologies in organic food and could help to better describe different target groups of consumers. 

## Data Availability

The data presented in this study are available on request from the corresponding author. The data are not publicly available due to privacy reasons.
